# The war between the immune system and the tumor - using immune biomarkers as tracers

**DOI:** 10.1186/s40364-024-00599-5

**Published:** 2024-05-30

**Authors:** Kai Yang, Rongrong Lu, Jie Mei, Kai Cao, Tianyu Zeng, Yijia Hua, Xiang Huang, Wei Li, Yongmei Yin

**Affiliations:** 1 Department of Oncology, The First Affiliated Hospital of Nanjing Medical University, Nanjing, 210029 P. R. China; 2 Gusu School, Nanjing Medical University, Nanjing, China

**Keywords:** Tumor immunity, Immune biomarker, Immunotherapy, Cancer

## Abstract

Nowadays, immunotherapy is one of the most promising anti-tumor therapeutic strategy. Specifically, immune-related targets can be used to predict the efficacy and side effects of immunotherapy and monitor the tumor immune response. In the past few decades, increasing numbers of novel immune biomarkers have been found to participate in certain links of the tumor immunity to contribute to the formation of immunosuppression and have entered clinical trials. Here, we systematically reviewed the oncogenesis and progression of cancer in the view of anti-tumor immunity, particularly in terms of tumor antigen expression (related to tumor immunogenicity) and tumor innate immunity to complement the cancer-immune cycle. From the perspective of integrated management of chronic cancer, we also appraised emerging factors affecting tumor immunity (including metabolic, microbial, and exercise-related markers). We finally summarized the clinical studies and applications based on immune biomarkers. Overall, immune biomarkers participate in promoting the development of more precise and individualized immunotherapy by predicting, monitoring, and regulating tumor immune response. Therefore, targeting immune biomarkers may lead to the development of innovative clinical applications.

## Background

As the third revolution in the history of drug therapy for malignant tumors, immunotherapy is regarded as one of the most promising anti-tumor therapies at present [1]. According to the immune surveillance theory, immunotherapy, which differs from conventional radiotherapy, chemotherapy, and targeted therapy, can leverage the anti-tumor immune system of patients to recognize and eliminate foreign tissues, such as tumors [2]. Additionally, it plays a crucial role in maintaining the equilibrium stage or potentially restoring the clear stage through reversing the escape stage, as per the tumor immunoediting theory, thereby effectively managing tumor progression over an extended period [3]. Due to its high universality, good effect on responders and low side effects, immunotherapy is no longer just a late-line treatment for patients with advanced tumors, but has gradually become the main means of tumor treatment, actively used in adjuvant or neoadjuvant therapy, and combined with other anti-tumor drugs such as targeting or anti-angiogenesis [4]. Due to the high side effects of chemotherapy drugs and low patient compliance, immunotherapy is gradually replacing chemotherapy drugs in front-line treatment in advanced lung cancer, leding to a new era of “chemo-free” treatment [5]. In addition, with the advancement of immunological knowledge and biological techniques, the emergence of “synthetic immunology” (primarily involving artificial manipulation and the creation of human immunity) has contributed to making immunotherapy more precise and effective [6].

However, there are still problems in immunotherapy: few patients respond to immunotherapy, and many of them have side effects mainly caused by drug resistance and immune-related adverse reactions (IRAEs). Meanwhile, the research of immunotherapy drugs is also mired in difficulties due to the lack of suitable targets [7]. In order to solve the above problems, we need to deeply study all aspects of immunogenesis and development of tumor to explore more suitable immune biomarkers as targets for monitoring and treatment.

Tumor immune biomarkers are biological features or indicators that can be objectively measured and used to evaluate tumor immune progression [8]. It is used to predict the efficacy and side effects of immunotherapy, monitor the immune response of tumors and provide targets for related drug research. Then we can screen out patients who are more suitable for immunotherapy, and may even make non-responders turn to responders or transform “cold tumors” to “hot tumors“ [9]. The treatment based on immune biomarkers breaks the concept of unified standardized treatment according to different tumor sources in the past, but carries out precise individual treatment according to the expression of markers [10]. In fact, various molecules (like receptors or cytokines), cells (of different types and proportions), microstructures (like tertiary lymphoid structures (TLS)), and even pathological features (like tumor cell death) involved in each stage of tumor immunity can be used as potential markers. However, attention should be paid to the extent of its contribution to the overall tumor immunity and the conversion possibility of clinical application [11]. Interestingly, in addition to naturally occurring ones during tumor immunity, biomarkers can also be artificially induced. For example, genetically engineered bacteria can specifically infect tumors while expressing corresponding antigens as biomarkers for immune cells to recognize tumors, and even produce substances similar to immune checkpoint inhibitors (ICIs) [12]. In addition to the exploration of the biomarkers themselves, the acquisition and detection means of immune biomarkers, the design of drug structure and the way of administration also affect the clinical application value of markers to a large extent [13].

Therefore, in order to search for immune biomarkers with high tumor specificity, good efficacy, and convenient clinical application, we updated and refined the rate-limiting aspects of the cancer-immunity cycle related to markers based on the remarkable progress of the past decade, particularly in terms of tumor antigen expression (related to tumor immunogenicity) and tumor innate immunity. The treatment of cancer is now gradually entering the “era of chronic disease management.” In addition to individualized treatment based on biomarkers, we also described comprehensive treatment, which includes the patient’s diet, exercise, and lifestyle habits. Finally, we focused on the clinical application and research progress based on tumor immune biomarkers.

### Tumor immunity and its markers

The heterogeneity of tumor (primary drug resistance, Darwinian natural selection) and its ability to adapt to survival pressures (secondary drug resistance, Lamarkian evolution) result in the failure of therapeutic strategies that act directly on the tumor due to adaptive changes in the tumor target [14]. No matter how the tumor mutates, it is considered foreign to the body, exhibiting the behavior and characteristics of abnormal cells. Therefore, regulating the patients’ own tumor immune response sthrough immune biomarkers to indirectly fight against the tumor is a therapeutic strategy of “responding to all changes with no changes”. However, there are two main ways for the tumor to suppress anti-tumor immunity to form “cold tumors”. On the one hand, tumors can disguise themselves as normal cells to reduce their immunogenicity and thus reduce the detection of the immune system. On the other hand, tumors arm themselves with immunosuppressive tumor microenvironment (TME) to impede immune effects [15].

### Initiation of tumor immune response

#### Tumor antigens are the basic biomarkers for the immune system to recognize tumors

Tumor antigens are tumor biomarkers that are abnormally expressed with the development and progression of tumors, mainly due to abnormalities at the gene level, but also due to abnormalities in the antigen synthesis process, such as post-transcriptional RNA splicing disorder or post-translational protein modification disorder [16]. In addition, it takes time for mutations to accumulate in normal somatic cells until they affect genes crucial for cell proliferation and death, eventually leading to tumorigenesis. During this time, normal somatic cells can also produce tumor antigens due to mutations in tumor-related genes, and even induce anti-tumor immunity (similar to autoimmunity) in non-tumor situations [17].

On the one hand, when affected by external influences such as physical, chemical or biological carcinogenic factors, or by internal influences such as spontaneous DNA replication errors, the cell will have gene mutations, which belongs to classical genetics. It is mainly manifested as the imbalance between proto-oncogenes and tumor suppressor genes [18]. Changes in gene sequences cause tumor cells to produce antigens that normal cells do not express, namely tumor-specific antigens (TSAs, also known as neoantigens), with high immunogenicity and individual heterogeneity [19]. It is worth noting that the genetic mutations that cause carcinogenesis can be divided into a few key driver genes and a large number of accompanying passenger genes. Theoretically, the greater the number of mutations, that is, the higher the tumor mutation burden (TMB), the more TSAs will be produced, although not all mutations associated with TMB-H will enhance the antitumor immune response [20]. In addition, the quality of the mutation also affected the production of TSAs, The study found that TSAs mainly came from the mutation of non-driver genes, while the mutation of passenger genes has a weak effect on the fitness of tumor cells [21]. Mutations in the genome have been found to contribute to tumor growth in 5–10% of patients [22].

On the other hand, abnormal gene expression may occur in cells, It is mainly manifested in the differences in the timing, spatial distribution, and level of gene expression compared to normal physiological or non-cancerous pathological processes [23]. Because there is no genetic sequence change, tumor cells will produce antigens that normal cells can also express, that is, tumor-associated antigens (TAAs), which have weak immunogenicity due to the formation of immune tolerance. TAAs include cancerous testicular antigen, differentiated antigen, overexpressed antigen and cancerous embryo antigen [24]. Notably, some TAAs can also be derived from classical genetic pathways, such as gene recombination that reactivates silenced promoters. Unlike other TAAs, some carcinoembryonic antigens reexpressed later still have high immunogenicity because the expression of normal embryonic proteins precedes the formation of immune tolerance [25].

Different from TAA, TSA is more tumor specific and is key to initiating the body’s anti-tumor immunity and T cell effector activation [26]. However, tumor antigens are not only biomarkers of mediated immunity or anti-tumor drugs targeting tumors, some tumor antigens promote the occurrence and development of tumors to a large extent. For example, some breast cancer cells overexpress human epidermal growth factor receptor 2 (Her-2), which enhances cell proliferation and differentiation mediated by growth factor signaling [27]. Therefore, for tumors, the balance between the survival promotion effect of tumor antigens and the anti-tumor immunity they provoke determines the tumor immunophenotype, including sensitivity and resistance to immunotherapy. Overall, tumors tend to hide TSA and masquerade as normal cells by expressing TAA, mediating immune escape [28].

In addition to the antigens spontaneously produced in the above cellular carcinogenic pathways, foreign microbial antigens expressed by non-carcinogenic pathways, such as viruses and bacteria, can also be used as tumor antigens. Microbial antigens activate anti-microbial immunity while destroying host tumor cells [29]. According to the state of the infected cell, microbial antigens can be divided into two categories. The one type of antigens arise from the process in which normal cells get infected and transform into tumor cells. Most of them are only related to the early stage of tumor development, while the later stage of tumor progression is related to the carcinogenesis pathway it induces. Other normal cells will also produce the same antigens after being infected. The other type of antigens is expressed after specific infection of tumor cells, which are mostly expressed by genetically engineered bacteria [30]. In recent studies, human endogenous retroviruses (HERVs) have been found to be more than the remnants of ancient retroviruses with low transcriptional activity. They can be reactivated by several factors, such as environmental carcinogenic factors, which may drive the expression of oncogenes and activate anti-tumor innate immunity by inducing viral defense pathways [31].

#### Tumor cell death is a biomarker of the release of tumor internal antigens and the initiation of immune cell infiltration

Immune cells must be able to recognize and contact tumor antigens in order to initiate tumor immunity. The immune system isn’t able to actively detect tumors. For one thing, immune cells patrol and supervise the body’s cells, that is lymphocyte recirculation, to increase the chance of contact with tumor antigens. But the cells involved in this recirculation are mainly memory T and B cells, whose primary role is to maintain long-term immunity rather than to initiate initial immunity [32]. For another, tumor cells can hide tumor antigens by resisting senescence and death, reducing and modifying the expression of major histocompatibility complex (MHC), and gradually differentiate into less immunogenic phenotypes with the progress of tumor immunoediting [2].

However, the mismatch between uncontrolled tumor proliferation and local resource supply results in the lack of available resources and the accumulation of metabolic wastes, such as hypoxia, glucose deficiency, lactic acid accumulation and oxidative stress. It inevitably leads to tumor cell senescence, damage, such as endoplasmic reticulum (ER) stress, and non-apoptotic regulatory cell death (RCD), especially immunogenic cell death (ICD) [33]. The death of tumor cells will release tumor internal antigens, adenosine triphosphate (ATP) and high mobility group protein 1 (HMGB1) and other immune stimulators to activate the tumor immune response. Similarly, dying tumor cells can also express damage-associated molecular patterns (DAMPs), such as ER chaperone calreticulin (CRT/CALR) and heat shock protein (HSP), which attract and activate innate immune cells by binding to pattern recognition receptors (PRRs) [34]. In addition, senescent tumor cells also secrete a cocktail of proinflammatory cytokines to form the senescence-associated secretory phenotype (SASP), which in turn regulates TME. Changes in the proportion of senescent cells in tumors before and after treatment have been recognized as one of the hallmarks of cancer [35].

Therefore, the death or injury of tumor cells is an important biomarker for the release of tumor internal antigens and the initiation of immune invasion. It is often used as a clinical target to develop various therapeutic strategies for inducing non-apoptotic RCD in tumors, such as ICD inducers [36]. However, this does not explain why healthy tumor cells in early TME can be recognized by the immune system when resources are sufficient, especially before the immune system applies the selective pressure to screen for lower immunogenic phenotypes. In this regard, recent studies have found that differences in epigenetic modification of the RNA-binding protein cold shock domain-containing protein E1 (CSDE1) can lead to high or low immunogenic heterogeneity in early neonatal tumor cells [37].

However, the death or injury of tumor cells does not always initiate an anti-tumor immune response, and may even suppress tumor immunity. Since cell death often occurs inside the tumor tissue, it is possible that the ‘corpse’ of the tumor is buried by the TME and cannot be detected by immune cells [38]. Even if the immune infiltration is activated, most tumor cells can hide their own antigens and pretend to be ‘innocent bystanders’. At this time, a large number of infiltrated immune cells can act as ‘scavengers’ to dispose of the ‘corpse’, with the process of antigen presentation being inhibited by rapid removal of DAMPs and tumor debris, which induce immune tolerance [39]. It is worth noting that the ‘corpse’ of the tumor can also cause the ‘pollution’ of TME. For example, potassium ions released after tumor cell death can inhibit the antitumor effect of effector T cells (Teff) [40]. Released prostaglandin E2 (PGE2) can also inhibit the activation of DAMPs on macrophages and dendritic cells (DCs) [41]. Similarly, HMGB1 released after iron death of tumor cells induces polarization of M2-type macrophages by binding to AGE [42]. Besides, the high expression of CD39 and CD73 in TME can convert immune-activated ATP into immunosuppressive adenosine, thus forming a negative feedback mechanism of adenosine energy axis conducive to tumor development [43]. On the contrary, the death of the; siblings’ can cause other tumor cells to be alert and enter a ‘state of combat readiness’. Similar to innate immune cells, tumor cells can also express PRRs such as toll-like receptors (TLRs) and P2 × 4 purinergic receptors to recognize DAMPs molecules such as ATP. Then they can activate signaling pathways such as NF-κB and mitogen-activated protein kinase (MAPK), which promote tumor cell proliferation and resist death. What’s more, it induces the production of various inflammatory factors and chemokines to regulate the differentiation and recruitment of immune cells [44].

### Biomarkers of tumor innate immunity

The role of innate immunity can be roughly divided into two categories. One is to attack tumor cells directly through phagocytosis, including natural killer cells (NK cells) and macrophages. The other group activates the second line of defense against tumors, namely adaptive immunity, mainly involving DCs through antigen presentation [45]. In order to counter the innate immune response, tumor cells can directly affect the camp of innate immune cells, the bone marrow, by inhibiting the maturation of DCs, granulocytes, and macrophages to eventually form and recruit immunosuppressive myeloid-derived suppressor cells (MDSCs). MDSCs can be classified as granulocyte/polymorphonuclear cells MDSCs (PMN-MDSCs, CD11b + CD14-CD15+/CD66b+) and monocyte MDSCs (M-MDSCs, CD14 + CD15-HLA-DRlo/-) [46]. It is worth noting that the recent study has also found tumors can induce CD45 + erythroid progenitor cells (EPCs) to transdifferentiate into erythroid derived myeloid cells (EDMCs), thereby supplementing MDSCs at the source [47].

#### Build the first line of defense against tumor

**Fig. 1:**
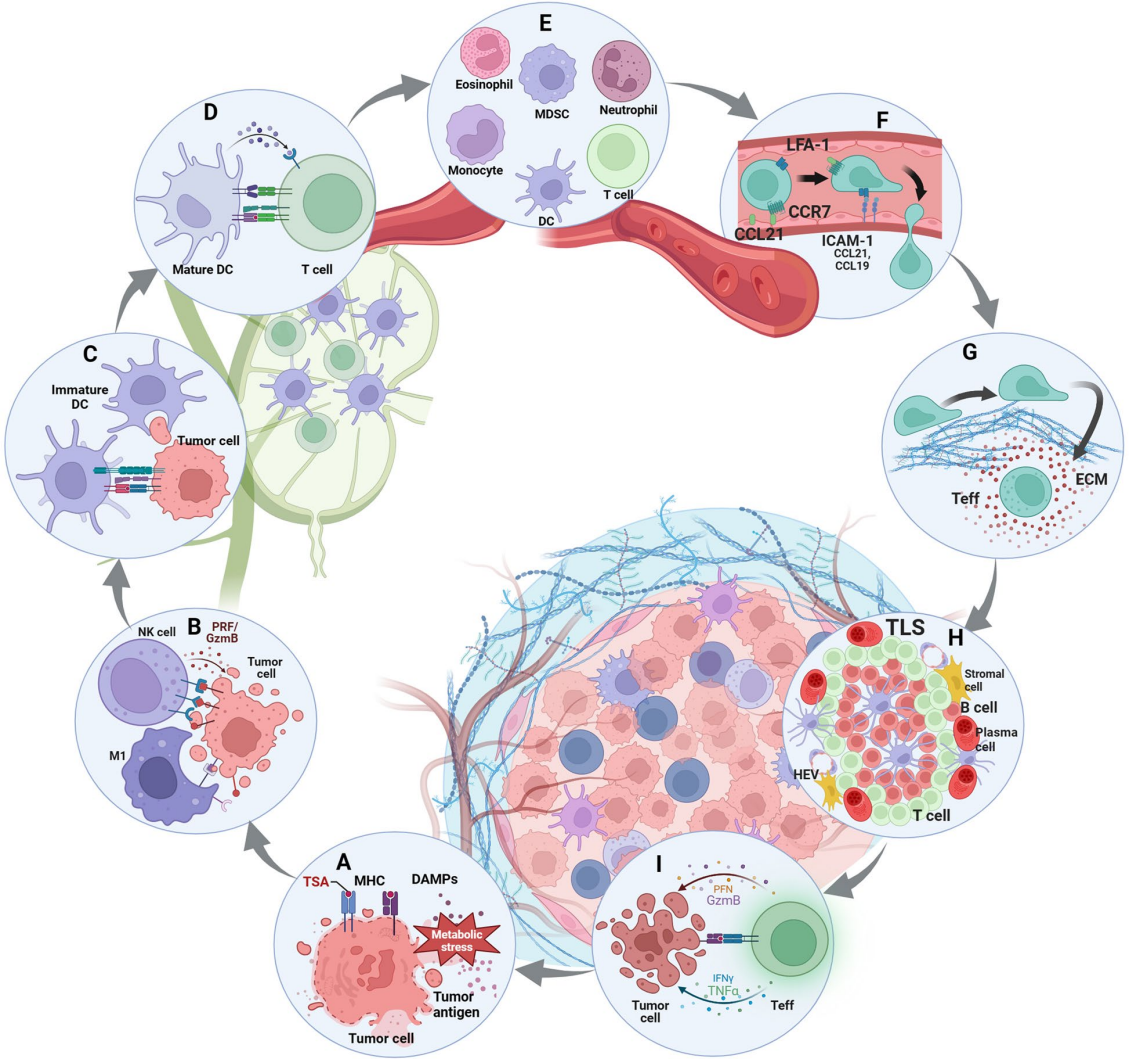
Various links in the development of tumor immunity. Initiation of the tumor immune response: **A** induce tumor cell ICD to release tumor antigens or promote tumor cell surface expression of pMHC to enhance immunogenicity. Building the first line of defense against tumors: **B** antitumor innate immune responses, including NK cells and macrophages, kill tumor cells through a balance of inhibitory and activating signals. Activated the second line of anti-tumor defense: **C** DC captured tumor antigens and migrated to TDLN and gradually matured. **D** DCs present antigen to T cells. The march of immune cells into the tumor stronghold: **E** chemokine-regulated immune recruitment in the peripheral blood circulation. **F** permeable malformed tumor neovascularization. **G** penetrate the tumor ECM with a rigid structure and immunosuppressive properties. Immune cells form the provisional command of the front line: **H** the formation of TLS. T cells exert effect: **I** T cells kill tumor cells through a balance of three signal**s**

**Fig. 2:**
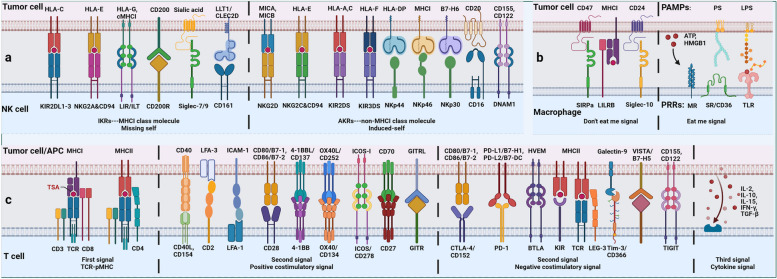
Balance between inhibitory and activation of cell-surface immune checkpoints. Anti-tumor innate immunity: NK cells regulate their activation state mainly through the balance of a series of activating killer cell receptors (AKRs, mainly recognizing non-MHCI molecules) and inhibitory killer cell receptors (IKRs, mainly recognizing MHCI molecules). Macrophages regulate their activation state mainly through the balance of a series of ‘eat me’ (DAMPs) and ‘don’t eat me’ (SIRPa) signals. Anti-tumor adaptive immunity: T cells regulate their activation state mainly through the balance of a series of TCR-pMHC, costimulatory molecules and cytokine signaling

NK cells are native cells with surface markers CD3-CD19-CD56 + CD16+ (CD3 is a marker for T cells and CD19 is a marker for B cells) and intracellular transcription factor E4BP4+. According to the difference of CD56 expression, NK cells can be divided into CD56dim, which mainly plays a cytotoxic role, and CD56bright, which mainly plays an immunomodulatory role [48]. Like T cells, subtypes such as regulatory NK cells, NK cell depletion, and tissue-resident NK cells have been identified [49]. Given the significant heterogeneity of NK cell infiltration in tumors, NK cell frequency predicts the efficacy of immunotherapies such as anti-programmed death receptor-1 (PD-1) [50]. Unlike Teff, NK cells do not express specific antigen-recognition receptors, but regulate activation state primarily through the balance of a series of activated killer cell receptors (AKRs) and inhibitory killer cell receptors (IKRs) signals (Figs. 1 and 2). NK cells do not require antigen presentation and have a broader spectrum, rapid, and safe (for allotransplantation) antitumor effect [51]. Therefore, AKRs and IKRs can be used as NK cell-associated immune checkpoint therapy (ICT) markers.

In general, non-MHCI molecules on the tumor surface activate NK cells after binding with AKRs while MHCI molecules suppress NK cells after binding with IKRs, but MHCI molecules can also activate AKRs [52]. In order to avoid the recognition of Teff, tumor cells usually down-regulate the expression of MHCI or have gene mutations of human leukocyte antigen (HLA) and β2 microglobulin (β2m). The loss of MHCI will mediate the killing of NK cells, that is, the ‘missing self’ effect, but that process can be disrupted. Due to the influence of TME, the number of NK cells infiltrated in the tumor was less, especially the subgroups with cytotoxic effects [53]. In addition, tumor cells can regulate the balance of AKRs and IKRs to keep NK cells in an inhibited state. For example, the non-classical HLA gene HLA-E has been shown to be highly expressed in various tumors and can provide inhibitory signals to NK cells and CD8 + αβT cells expressing NKG2A/CD94 [54].

Macrophages in tumors are mainly derived from circulating monocytes and tissue-resident macrophages, both of which form tumor-associated macrophages (TAMs) under the influence of TME [55]. In the early stage of immune response, like pro-inflammatory M1 type, TAM mainly plays a killing role, with high expression of CD80, CD86, MHCII, inducible nitric-oxide synthase (iNOS) and CD68 and is dependent on glycolysis. In the later stage of immune response, like anti-inflammatory M2 type, TAM mainly plays an immunosuppressive role, highly expressing CD206, CD204, vascular endothelial growth factor (VEGF), CD163 and arginase (Arg-1), and relying on fatty acid oxidation (FAO) [56]. The study has shown that cell frequency of TAMs is related to response to tumor immunotherapy [57]. Similar to NK cells, TAM regulates activation state mainly through the balance of a series of ‘eat me’ and ‘don’t eat me’ signals which act as a macrophage-related ICT marker. For example, DAMPs expressed by tumor cells convey a ‘eat me’ signal to macrophages, but MHCI expressed by tumor cells conveys a ‘don’t eat me’ signal [58].

#### Activate the second line of anti-tumor defense

Only DCs can activate initial T cells (Th0) in tumor draining lymph nodes (TDLNs) to initiate an adaptive immune response de novo [59]. Tumor of immature DCs have high expression of PRRs but low expression of MHC, co-stimulatory and adhesion molecules so that they have strong antigen uptake and processing ability but weak ability to present antigen. In the process of migration to TDLN, DCs gradually mature and exhibit receptor expression and function opposite to the immature state [60]. Notably, tumors inhibit the maturation of DC through immunosuppressive TME, specifically tumor extracellular matrix (ECM) related components. For example, the presentation of tumor antigens can be inhibited by mechanisms such as DC-dependent T cell activation induced cell death (AICD) [61]. The maturation of type 1 conventional dendritic cells (cDC1) was found to be interfered with by NF-κB and specific inactivation of interferon regulatory factor 1 (IRF1) [62]. More importantly, tumor-derived immunosuppressive factors (such as transforming growth factor β (TGF-β)) and tumor cells with lymph node metastasis can reshape the TDLN microenvironment and directly affect the production of Teff [63].

Unlike the state of individual cell types, the collection of cells with functional connections, that is, the immune archetype, better reflects the intrinsic connections and interactions of tumor immunity [64]. For example, cDC1 mainly supports the effects of proliferative tumor-antigen specific TCF-1 + CD8 + T cells and is regulated by chemokines secreted by NK cells [65]. Type 2 conventional dendritic cell (cDC2) mainly supports the effects of CD4 + T cells and is inhibited by their derivative subtype regulatory T cells (Tregs) [66]. Contrary to conventional wisdom, the recent study has shown that cDC1 can activate CD4 + T cells through MHCII cross-presentation of tumor antigens. In turn, CD4 + T cells can also promote cDC1 function through CD40 signaling [60]. In addition, a subpopulation of DCs characterized by immunomodulatory genes, namely mature regulatory dendritic cell (mregDC) rich in immunomodulatory molecules, are associated with uptake of dead tumor cells and inhibit the role of cDC1-activated T cells in TDLN [67]. Therefore, all the molecules, cell types and TDLN microenvironment involved in the antigen presentation process of DCs and T cells can be used as biomarkers of immunotherapy.

### Biomarkers of tumor adaptive immunity

The strategies of innate and adaptive immunity to recognize tumors are different. Innate immunity mainly recognizes the absence of normal cell biomarkers through the ‘missing self’ effect while adaptive immunity mainly recognizes the appearance of abnormal tumor biomarkers by binding T cell receptors (TCRs) to antigenic peptide-MHC (pMHC) [68]. Among them, tumor adaptive immunity mainly relies on T cells. But for tumor humoral immunity, on the one hand, tumor-specific antibodies can activate complement-dependent cytotoxicity (CDC), antibody-dependent cell-mediated cytotoxicity (ADCC), and antibody-dependent cell-mediated phagocytosis (ADCP) of NK cells and macrophages, and even block some tumor-promoting growth factor receptors [69]. On the other hand, tumor-specific antibodies cannot target internal tumor antigens when the expression level of tumor surface antigens is low [70]. Certain boosting antibodies or tumor-targeting monoclonal antibodies (mAbs) can even inhibit the cytotoxic effect of NK and T cells by promoting the expression of PD-L1 and indoleamine 2, 3-dioxygenase (IDO). They can also promote tumor metastasis by binding to cell adhesion molecules [71].

#### Immune cells enter the tumor base camp

Although current immunotherapy mainly focuses on remodeling the local TME, more and more studies have begun to focus on the systemic immunity beyond tumors, including TDLN, bone marrow, and peripheral blood circulation. The study has found that the frequency of immune cells and the level of immune molecules in blood circulation can be used as potential markers for immunotherapy [72]. In addition, Teff in TME in response to ICT are mainly derived from the peripheral input of T cell precursors of exhaustion (Tpex) in TDLN rather than from local T cell exhaustion (Tex), indicating that immunotherapy not only regulates the local TME, but also changes the systemic immunity of whole body [73].

With the function of the corresponding chemokines, immune cells are released from the TNDL and bone marrow into the bloodstream and directed to the site of the tumor immune response. But chemokine secretion in TME is often altered [74]. Since different immune cell subpopulation has different chemokine receptor expression patterns, abnormal expression of chemokines may regulate the differentiation, recruitment, homing and recycling of lymphocytes according to the needs of tumor survival or therapeutic intervention [75]. In addition, chemokines are involved in tumor angiogenesis, proliferation, and invasiveness by directly targeting non-immune cells in TME, such as tumor, stromal, and vascular endothelial cells. Therefore, chemokines are important biomarkers of tumor immunity and valuable therapeutic targets [76].

However, the process by which immune cells infiltrate tumor is hindered in many ways. The first barrier that immune cells encounter in the circulation of the blood is the endovascular glycocalyx formed by the ECM. The malformed tumor neovascularization system prevents the immune cells from entering the tumor [77]. Depletion of the Rgs gene has been found to normalize tumor vasculature, thereby promoting immune cell infiltration [78]. Different from the classical endothelium-dependent tumor angiogenesis pathway, tumor vasculogenic mimicry (VM) is a novel tumor microcirculation model independent of the body’s endothelial cells. The vascular pathway is constructed by the tumor cells themselves. Although there is a lack of direct studies to prove the effect of VM on tumor immunity, it can be speculated that the absence of endothelial cells impedes the endothelium-dependent adhesion and migration of immune cells, thereby hindering immune infiltration and distribution within the tumor. More importantly, rigid structure and immunosuppressive tumor ECM, especially collagen secreted by cancer-associated fibroblasts (CAFs), not only hinder the infiltration of immune cells. They also reduce the release of tumor antigens, interfere with antigen presentation and affect the effect of T cells, and even prevent the penetration of anti-tumor drugs [79]. However, ECM, as a protective shell of the tumor, can also hinder the outward progression and metastasis of the tumor, so the dissolution of ECM is one of the signs of tumor metastasis [80].

#### Immune cells form a temporary command line

In order to combat the immunosuppressive TME, immune cells in tumors do not fight alone but form a systematic team battle and intratumoral immunity cycle. This is evident in the formation of a densely populated area with MHCII high expression APC and CD8 + T cells, known as the APC niche, based on the immune archetype [81]. They may also form more types of lymphoid structures that regularly gather immune cells, known as TLS. The APC niche and TLS not only reflect the number and location of immune cell infiltration in the tumor, but also reflect the heterogeneity of its spatial distribution and the interaction between functions [82]. The study has found that the APC niche is associated with producing an effective and long-lasting immunotherapy response, but it is unclear whether the APC niche is an early stage of TLS. Similarly, the study has found that tumor-specific CD8 + T cells aggregated in TLS are rarely functionally exhausted and exhibit typical memory characteristics [83]. The study has also found that TLS mediates B cell maturation, plasma cell differentiation, and antibody formation [84]. Therefore, the development of TLS is associated with improved ICT efficacy, which can enhance immune infiltration and effect, and generate anti-tumor immune sites [81]. . Therefore, the APC niche and TLS are important markers for immunotherapy. However, TLS is also affected by tumor-produced cytokines and metabolic factors, such as TLS-associated Tregs, regulatory B cells (Bregs), interleukin-10 (IL-10), and enhanced antibodies, which lead to the formation of immunosuppressive TLS and macrophage or NK-cell-dependent apoptosis [85].

#### Process and outcome of T cell effect

Diffrent from the classical mode of activation for acute bacterial or viral infections, T cell activation of tumor can be divided into two stages: initial activation of cDC antigen presentation in TDLN and subsequent effector activation within the tumor [86]. Among them, effector activation can be roughly divided into three signals: TCR-pMHC, co-stimulatory molecules and cytokines [86]. Interestingly, the effect of T cells is not limited to single one cell, but is accumulated with the number of cells and the duration of action, that is, additive cytotoxicity. Particularly in solid tumors, sublethal injury events delivered by multiple T cells to a single tumor cell mediate effective tumor killing through time-dependent integration [87].

Most current studies focus on CD8 + T cells and tumor-constitutionally expressed MHCI, but the cytotoxic effects of CD4 + T cells and tumor-induced expression of MHCII also deserve our attention. The study has shown that interferon-γ (IFN-γ) induces tumor cells to express MHCII through MHC class II transactivator (CIITA), which is recognized and killed by cytotoxic CD4 + T lymphocyte (CD4 + CTL) [88]. Among them, class I MHC-restricted T-cell-associated molecules (CRTAM) can be used as early biomarkers of CD4 + CTL [89]. The effects of CD4 + CTL require external stimulation to be maintained [90]. Unlike MHCI, MHCII can bind to a higher diversity of antigenic peptides, thereby increasing the likelihood of CD4 + CTL recognition of TSA [88]. More importantly, the study found that MHCI and MHCII are independently regulated in tumor immunity, so their expression may have independent implications for immunotherapy [91].

Tumor cells can evade T-cells’ killing function in a variety of ways. In TDLN, immature DCs, upregulated TGF-β and down-regulated IL-2 can maintain T cells in quiescence. The transcription factor FOXO1 and its induced Kruppel-like factor 2 (KLF2) as well as V-domain Ig suppressor of T cell activation (VISTA) were highly expressed in static T cells [92]. However, tumor cells downregulate the expression of MHCI to induce the loss of TCR-pMHC signal, thus keeping T cells ignorant. Unlike quiescence, ignoring T cells can not specifically recognize tumor antigens [93]. However, even if the TCR-pMHC signal can be activated, the tumor can still maintain T cell anergy by downregulating positive co-stimulatory molecules [94]. The expression of IL-12, IFN-γ and tumor necrosis factor (TNF) was significantly decreased by anergic T cells. Only in the absence of tumor antigen stimulation can anergic T cells gradually resume their functional response [95]. However, interestingly, even in the absence of external activation from tumor cells, T cells can activate CD28 costimulatory signals by cis-B7:CD28 interactions at invaginated synapticmembranes, that is autologous signaling, thereby enhancing their ability to attack tumors [96].

In addition to down-regulating positive costimulatory molecules, tumor cells can also up-regulate the expression of negative costimulatory molecules. This helps maintain T cells in a state of depletion under the influence of stimulatory factors in TME such as chronic TCR signaling [97]. Genetic analysis revealed extensive chromatin remodeling and a close correlation with transcriptional regulatory factor TOX in Tex [98]. Although Tex overlaps with anergic T cells in the expression of inhibitory receptors, anergic T cells appear primarily early in immune response while Tex is produced late from Teff [99]. Based on the expression of Ly108 (representing transcription factor TCF1) and CD69, Tex is divided into four stages, namely, T cell exhaustion progenitors 1 (TexProg1), T cell exhaustion progenitors 2 (TexProg2), T cell exhaustion intermediate (TexInt) and T cell exhaustion terminally (TexTerm). Starting from TexInt, Tex gradually loses the expression of TCF1, so anti-PD-1 treatment can restore the function of Tex in the first three stages, which may be related to the fact that Tex has not completed the secondary epigenetic regulation [100]. However, not all T cells will be exhausted and lose function. The study has found that some T cells can be pluripotent and regenerative after depletion, that is Tpex. Unlike Tex, Tpex can express CD62L, TCF1, ID3, CXCR5, Ly108 and the transcription factor MYB [101]. Tpex is essential to maintain the Tex library and facilitate the response of ICT. Further study showed that cDC1 insulates Tpex from persistent tumor antigens through the action of pMHCI-TCR to prevent its further depletion [102]. TGF-β has also been shown to be closely related to the immune signal of depletion [103].

In addition, activated T cells can also be induced to develop AICD (peripheral deletional tolerance/death) in the absence of growth factors (such as IL-2) by apoptosis factors such as FasL, TNF and TNF-related apoptosis-inducing ligand (TRAIL) in TME [104]. It was found that the mutual inhibition of long non-coding RNA (lncRNA) NKILA and nuclear foctor-κB (NF-κB) can regulate the sensitivity of T cells to AICD [105]. In conclusion, multi-dimensional integrated T cell profiling, including TCR antigen specificity, sequence analysis, T cell phenotype, activation/exhaustion state, and clonal proliferation, is essential for the monitoring of T cell-based cancer immunotherapy [106].

### Emerging important factors affecting tumor immunity

#### Competition for resource supply: metabolism-related biomarkers

**Fig. 3:**
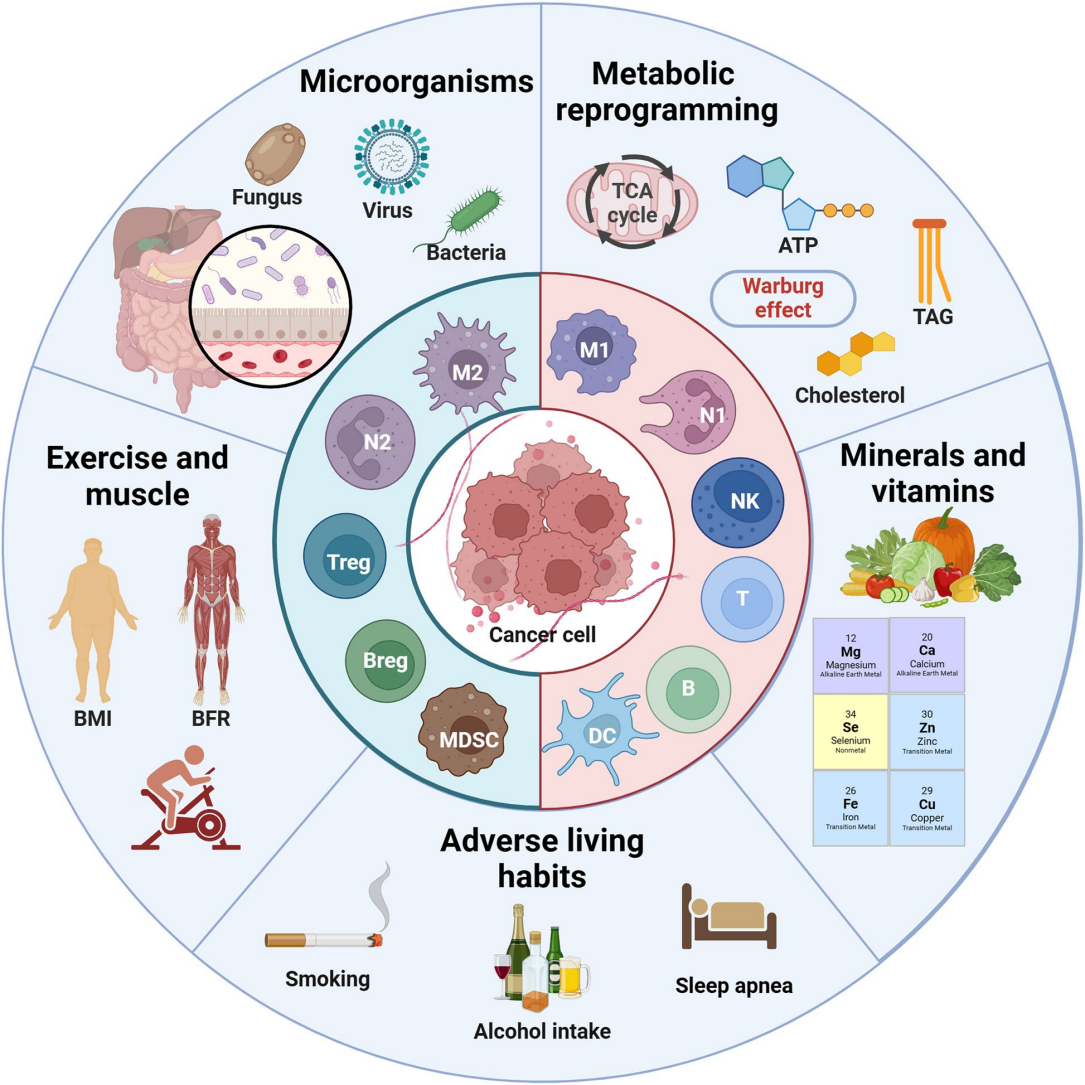
Important off-field factors affecting tumor immunity. In addition to tumor immunity itself, metabolic reprogramming (especially lipid metabolism) of tumor and related immune cells, as well as specific trace elements and vitamins affect the energy competition between cells at the metabolic level. As a new field of tumor immunity, tumor microbes and the microenvironment they create affect various aspects of tumor immunity, such as tumor immunogenicity, antigen presentation, ICD, metabolism, and ICs. In addition, exercise and bad habits also play an auxiliary role in tumor immune response that cannot be ignored. The above factors can be used as potential targets for regulating TME and improving immunotherapy

Tumor and immune cells adapt to various stress challenges in TME, such as hypoxia, nutritional competition (deficiency of glucose and key amino acids), acidic environment, and oxidative stress (lipid peroxidation), by changing their own metabolic patterns, that is, metabolic reprogramming [107]. The enhancement of aerobic glycolysis is an important sign of T cell activation and anti-tumor effect. But tumor cells rely on anaerobic glycolysis, that is “Warburg effect”, leading to cell competition for glucose [108]. In addition, tumors of non-gluconeogenic tissues, except for liver and kidney cancer, are able to compensate for glucose deficiency through partial activation of the gluconeogenic pathway [109]. In addition to being an alternative energy source, enhanced lipid metabolism promotes tumor invasion and metastasis, which is usually manifested as an increase in the proportion of unsaturated fatty acids (especially monounsaturated fatty acids) on the cell membrane and the accumulation of intracellular cholesterol. However, lipid metabolism promotes the transformation of immune cells to a protumor phenotype and inhibits the function of anti-tumor immune cells [110]. Similarly, tumor cells and immune cells compete for key amino acids, such as glutamine (Gln), which is essential for cell proliferation, metabolite production, and fatty acid synthesis [111]. In addition, the overexpression of IDO in tumor cells promotes the essential amino acid tryptophan (Trp) to catalyze kynurenine (Kyn). It leads to both the suppression of anti-tumor T cells caused by Trp deficiency and the activation of tumor-promoting immune cells mediated by Kyn accumulation [112]. Notably, some metabolism-regulating drugs can be used as concomitant drugs to promote tumor immunotherapy. For example, statins inhibit their ability to stabilize the expression of PD-1/PD-L1 on the cell membrane by blocking the synthesis of cholesterol [113]. In addition, metformin promoted the transformation of TME to an antitumor phenotype by increasing the number of CD8 + T cells while decreasing the expression of M2 macrophages (Fig. 3) [114].

### Supply of resource supplements: trace element and vitamin related biomarkers

Nutritional status is closely related to immune homeostasis, especially for cancer which is a wasting disease. The changes in the levels of related trace elements and vitamins in the body will affect the tumor immune response [115]. Carcinogenic trace elements are mostly heavy metal elements, such as arsenic, chromium, nickel, beryllium, cadmium and lead, while anti-cancer trace elements, such as selenium, magnesium, zinc, molybdenum, iron, calcium and copper. For example, selenium compounds protect cell-membrane structures and promote antitumor immune responses, primarily through antioxidant effects, particularly the clearance of lipid peroxides [116]. Similarly, Ca²^+^, Fe²^+^ and Cu²^+^ also affect the REDOX balance and mediate Ca²^+^ overload, ferroptosis and cuproptosis of tumor cells to activate anti-tumor immune responses [110]. In addition, zinc also promotes lipid metabolism and decreases cellular lipid supply and accumulation [117]. It is worth noting that trace elements have a relatively strict proportion in the body. High or low content or improper proportion of each element will affect tumor immunity. For example, excessive iron will also lead to oxidative stress of immune cells, resulting in ferroptosis and inhibition of immune function [118]. In addition to trace elements, vitamins also affect tumor immunity. For example, high-dose vitamin C can increase T-cell infiltration and improve the efficacy of ICT [119]. Similarly, dietary supplementation with vitamin E could enhance patient response to ICT by increasing tumor antigen presentation and activating T cell-mediated antitumor effects [120]. However, dietary ingredients and nutritional supplements are diverse and complex in composition, and their modulation of antitumor immune response and contribution to immunotherapy remain to be explored.

### The third party of tumor immunity: microbial-related biomarkers

The influence of microorganisms on tumors can be roughly divided into two aspects. For one thing, intestinal microorganisms indirectly affect the progression of tumors mainly through metabolic pathways. For example, gastrointestinal cancer and liver cancer may be regulated by the metabolites of intestinal microorganisms such as bile acids [121]; In breast cancer, intestinal microorganisms may interfer with steroid metabolism and lead to changes in estrogen spectrum [122]. Even brain tumors are thought to be influenced by gut microbes via the gut-brain axis [123]. For another, the microbiota within tumors has also been shown to influence tumor progression and therapeutic response. For example, increased intratumoral microbiota can lead to mutagenesis in tumorigenesis, modulation of carcinogenic pathways, or alterations in the host immune system [124]. The study has found that intestinal flora can predict the efficacy, change the metabolism of immunotherapy drugs, and affect the bioavailability and efficacy of drugs in the host body [125]. In general, patients with higher diversity and more stable composition of intestinal flora have better ICT efficacy [124]. Besides, opportunistic fungi can also influence the immune response through the fungus-sensing protein Dectin-1. Therefore, inhibiting bacteria may impair tumor immunity while inhibiting fungi may enhance immunotherapy efficacy [126]. However, given the diversity of microbiome and the dynamic changes in microenvironment, as well as the complex links between the microbiome and genetics, environment and diet, the complex interactions between the microbiome and tumor immunity need to be further studied with standardized measures [124].

### Auxiliary regulation of tumor immunity: exercise-related biomarkers

Exercise is an important part of comprehensive treatment of cancer and is related to the amount, time, frequency, and mode of exercise. The study found that moderate-to-vigorous intensity aerobic exercise or late morning aerobic exercise was more effective in improving metabolism and lipolysis, while there was no significant difference in the risk of death from cancer between regular and concentrated daily exercise [127]. Of note, the effects of exercise in reducing tumor mortality and delaying tumor progression, which is difficult to reverse existing tumors, are achieved primarily by promoting antitumor immunity [128]. Specifically, exercise enhances the differentiation and recruitment of anti-tumor immune cells by stimulating sympathetic nerves, regulating endocrine levels, accelerating metabolism and even improving mood, which helps relieve immunosuppressive factors such as hypoxia, oxidative stress and high lipid in TME [127]. For example, aerobic exercise can inhibit the growth of pancreatic cancer in mice by activating the immune system, especially IL-15Rα + CD8 + T cells [129]. As an indicator of exercise performance, the body’s muscle mass can be used as a potential marker of tumor immunity, and the study has also considered sarcopenia as a marker of poor prognosis of ICT [130]. The newly generated muscle will secrete many hormones and growth factors during exercise and contraction, which are collectively called myokine [131]. IL-4/6/7/8/15 helps to maintain the balance between pro-inflammatory and anti-inflammatory mediators and promote the differentiation, recruitment and effect of anti-tumor immune cells. In addition, the increase in actin in the circulation caused by exercise helps to activate the immune system [132].

### Effect of bad living habits on tumor immunity

Unhealthy lifestyle habits, such as smoking, drinking and staying up late, not only have carcinogenic effects, but also have immunosuppressive effects. Smoking or second hand smoke can lead to a variety of cancers [133]. Interestingly, smokers have a better effect on ICT than non-smokers. The further study has found that cigarette smoke induces the overexpression of PD-L1 through aryl hydrocarbon receptor, which leads to tumor immune escape and sensitivity to ICT [134]. However, some studies have found that smokers’ tumors highly express tissue resident memory T cells (TRM), which mediates immune escape and is insensitive to immunotherapy by exerting immune pressure on tumors [135]. Smoking also raises risk for colorectal cancer (CRC), especially in people with low T-cell responses. Similarly, alcohol use, even in moderation, reduces antitumor immune surveillance by mediating DNA damage (acetaldehyde), affecting the metabolism of micronutrients (e.g., folate, vitamins B12, B6, and A), or generating large amounts of free radicals [136]. In addition, alcohol was found to promote PD-L1 expression on CRC cells through the induction of aldehyde dehydrogenase 2 [137]. Similarly, it has been demonstrated that the disruption of circadian clock caused by regular stay up will greatly destroy the proportion of immune cells and induce immunosuppressive TME, which will significantly reduce the ability of immune cells to destroy cancer cells and promote the rapid growth of tumors [138].

### Clinical application of tumor immune biomarkers

Tumor immune biomarkers can be roughly divided into two categories. One is tumor-related biomarkers, mainly tumor antigens, which are used to directly target tumor cells. The other is immune-related biomarkers, mainly immune checkpoints (ICs), which are used to modulate the state of the tumor immune response. Tumor specificity largely determines the clinical application value of tumor immune biomarkers [139].

It is imperative to explore the optimal utilization of biomarkers to enhance their clinical applicability. This encompasses the identification and assessment techniques of markers, the design of drug structures, and the methods of drug delivery. Markers from invasive examination such as biopsy specimens can only reflect the static state of part tumor tissues at a certain point in time due to the heterogeneity of tumor tissues, so the results may be biased or not time-effective [140]. Although the markers from non-invasive examinations such as blood are convenient to dynamically reflect the overall situation of the tumor in real time, the results are difficult to detect or not accurate enough due to low expression level or many other interfering factors [141]. To better detect the expression of PD-L1, new liquid detection methods are being studied, including soluble PD-L1 (sPD-L1) [142], circulating tumor PD-L1 (PD-L1*CTCs) and exosomal PD-L1 [143]. Other liquid detection methods for tumors based on DNA differences in mature red blood cells are also under the research [144]. Different from the traditional “pathological biopsy + immunohistochemistry,” which is subject to the dynamic changes and heterogeneity of the target, immuno-positron emission tomography (immuno-PET) is a novel diagnostic technology that is highly specific, sensitive, non-invasive, and provides real-time dynamic imaging in vivo [145]. By labeling monoclonal antibodies with positron nuclides, immuno-PET combines the targeting specificity of antibodies with the high sensitivity of PET. This approach provides information on tumor immune responses across focal areas, treatment stages, and tumor progression directly by monitoring immune biomarkers such as CTLA-4, PD-1/L1, HER2, and MHC molecules [146, 147].

The drug structure about tumor antigen is mostly similar to antibody drug conjugate (ADC), that is, warhead (such as antibody, TCR or chimeric antigen receptor (CAR) targeting tumor antigen) plus ammunition (such as toxin, drug or immune cells killing tumor cells) and the connection structure of the two. The influencing factors include lysability, solubility, drug antibody coupling ratio (DAR) and bystander effect [148]. The drug structure around ICs is dominated by antibodies, which can be divided into active and blocking types. To increase tumor permeability and reduce immunogenicity, only the Fab segment can be retained, but the Fc segment can mediate the ADCC effect [149].

Regarding drug administration, considering the attributes of chronic tumor diseases and the requirement for prolonged drug usage, the future trajectory for antitumor drugs lies in subcutaneous intratumoral injection or even oral administration (nano-strategies) [150]. Furthermore, the integration of bionic nanoparticles with cell membrane coatings (such as erythrocyte membranes and engineered tumor membranes) enhances the tumor-targeting capability and biocompatibility of drugs, building upon the inherent selective permeability and vascular retention properties of conventional nanomaterials [151]. Similarly, engineered bacteria combined with quorum sensing effects can also help achieve targeted tumor killing [152].

It is important to acknowledge that the combination of different biomarkers can exert better anti-tumor effect. For instance, the combination of ICD inducers and ICT can stimulate immune infiltration while preserving their anti-tumor capabilities [153]. ICIs combined with immune adjuvant, such as IL-15 superagonist, can synergistically enhance anti-tumor immune activation [154]. Additionally, the integration of ICIs with TGF-β antibodies or ECM inhibitors facilitates drug penetration into the tumor [155]. Biomarkers that target both immune and tumor cells can aid immune cells in approaching tumor cells and initiating the cytotoxic effect. The redirection of engineered immune cells by bispecific antibodies (BsAbs) can reverse the off-target and drug resistance of cellular immunotherapy [156].

### Treatment strategies based on tumor antigens

**Fig. 4:**
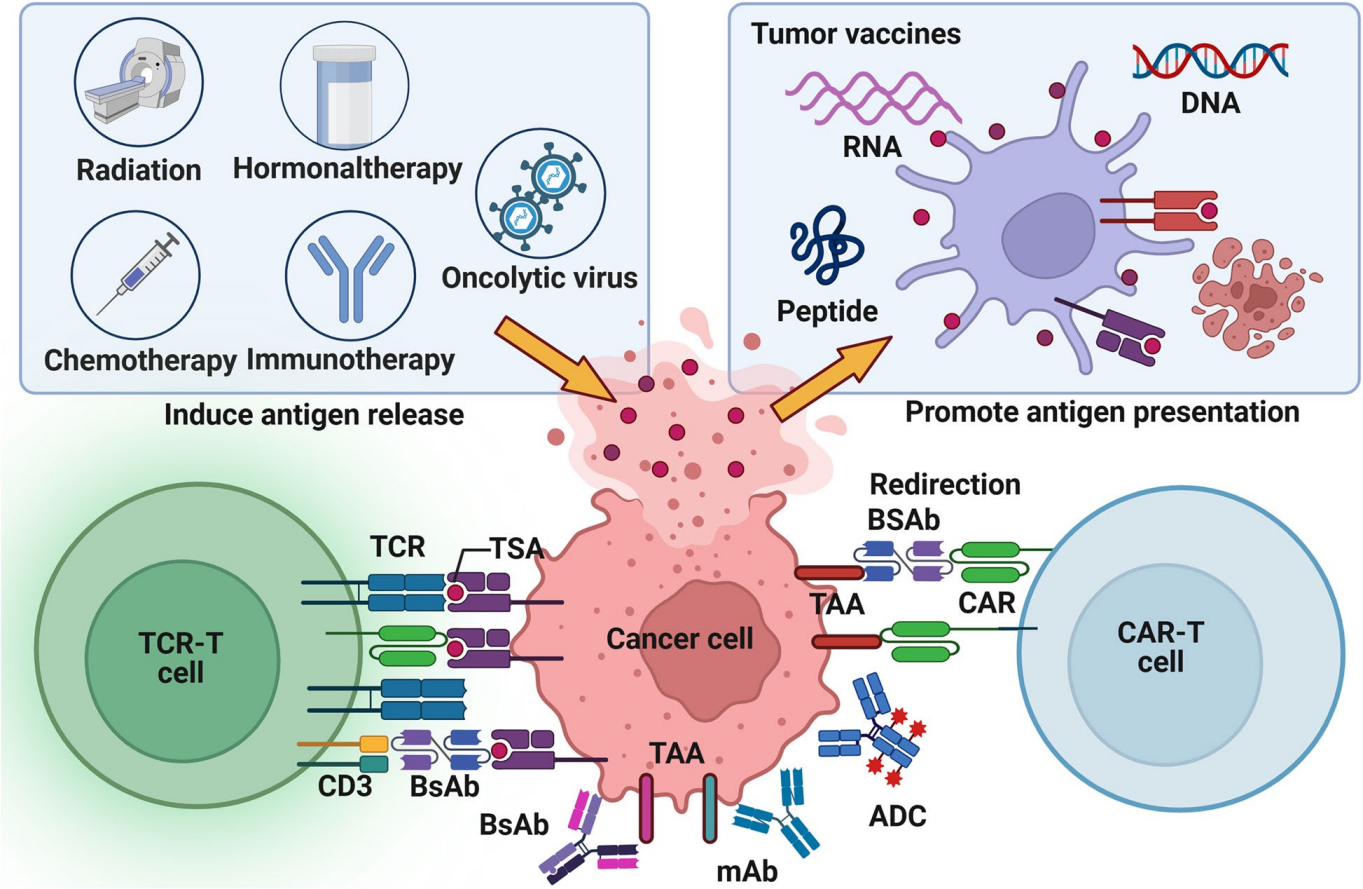
Tumor antigen-based treatment strategies. Tumor antigen-based therapies encompass tumor vaccines, ACT, tumor antibodies, and inducers of ICD. Tumor vaccines emulate the mechanism through which tumor cells release or express tumor antigens, thereby initiating and augmenting anti-tumor T cell immune responses. These vaccines offer many advantages, including eliciting a broad spectrum of anti-tumor immunity, exhibiting high variability, and demonstrating robust efficacy in overcoming tumor heterogeneity. Compared to established vaccines for infectious diseases, tumor vaccines are relatively straightforward and cost-effective to prepare, except for DC vaccines. However, their utilization in clinical settings remains infrequent. Adoptive cell therapy replicates the procedure of screening and amplifying anti-tumor immune cells, resulting in a substantial enhancement in both quantity and efficacy of these cells. The advantages of ACT in anti-tumor immunity encompass robust and enduring response, prompt initiation, heightened specificity, and potent capacity to overcome immunosuppression. Thereby, ACT circumvents the issue of adverse effects associated with excessive immune stimulation. Nevertheless, certain challenges persist, including limited scope of anti-tumor immune response, diminished variability, inadequate ability to surmount tumor heterogeneity, arduous preparation, protracted process and high cost. Synthetic tumor-targeting antibodies emulate the functional mechanism of naturally occurring antibodies in vivo, thus labeling tumor cells, promoting ADCC effect, blocking tumor-promoting receptor signaling, connecting target cells with killer cells, and carrying anti-tumor substances. Tumor antibodies have the advantages of strong anti-tumor immune responses, high specificity, low side effects and relatively simple preparation, especially for hematological tumors. They account for a large part of the researches and development of anticancer drugs and some of them have entered clinical application. ICD inducers promote tumor production of DAMPs to activate anti-tumor innate and adaptive immune responses. The current clinical application of ICD inducers tends to combine them with immunotherapy or targeted drugs for anti-tumor therapy, and to develop new drug delivery methods

In comparison to TAA, TSA (especially the epitopes) has higher immunogenicity, closer relationship with tumor and more stable expression, so it is not easy to induce immune tolerance, autoimmune response and drug resistance [157]. Therefore, TSA has become a highly potential biomarker in tumor antigen-based therapeutic strategies. Due to the strong heterogeneity of TSA, it is difficult to determine the inevitable relationship between a certain mutation type and TSA, and the number of TSA has been identified is far less and more difficult to apply than TAA [158]. Therefore, we still need to actively search for more TSA shared in tumor patients, such as TSA generated by KRAS G12D2 mutation [159]. An evaluation of the priority of tumor antigens at different expression sites shows that, based on clinical effect, immunogenicity, specificity, tumor correlation, expression level and positive rate, and cell expression distribution, internal tumor antigens (mainly TSA) are more clinically useful than tumor surface antigens and soluble secreted antigens (mainly TAA) [160]. However, it is difficult for most antigen-based treatment strategies to directly target antigens inside tumor cells [158]. Thus, inducing the death of tumor cells contributes to the release of antigens within the tumor and contact with immune cells (Fig. 4).

#### Targeting tumor antigens: tumor surface antigens and MHC-bound internal antigens

The therapeutic strategies targeting tumor antigens, especially TSA, mainly include tumor vaccines, adoptive cell therapy (ACT), and tumor antibodies [158]. For relatively easy-produced and inexpensive tumor vaccines, although the development of preventive vaccines has made significant achievements, most therapeutic vaccines are limited to phase III clinical studies due to low immunogenicity [161]. Therefore, DC vaccines with high immunogenicity and active antigen presentation may be an important direction for future development, but it is still difficult and expensive to produce [162]. Traditional tumor vaccines mainly focus on improving the cellular activity of CD8 + T cells, while the screening of tumor antigen dominant CTL epitope peptides is helpful to find new immune active targets that synergistically activate CD8 + T cells, NK cells and DC [163].

Different from the directly expressed tumor surface antigens, MHC can bind to the intracellular concentrated and processed tumor internal antigens, and express them on the cell surface [164]. Unlike CAR-T cell therapy and tumor antibodies restricted to tumor surface antigens, TCR-T cell therapy utilizes the immune surveillance mechanism to more sensitively recognize a wider range of tumor internal antigens with low levels of variation and thus play a better anti-tumor effect [26]. However, the adaptive regulation of MHC expression level and the individualized restriction of MHC make it difficult for TCR-T to be developed as universal as CAR-T/NK cell therapy, and it is not easy to recognize lipid or carbohydrate tumor-related substances [165]. In addition, both ACT and tumor antibodies are susceptible to the interference of soluble targets and are mistakenly activated in advance (Table 1) [166].

**Table 1:** Therapeutic strategies targeting tumor antigens

Classification			Advantages	Disadvantages	Development	Clinical trials
Cancer vaccine (active immunization)	Tumor antigen components	Peptide/protein vaccines (anti-idiotypic antibody vaccines and heat shock protein-peptide complex (HSPPC) vaccines)	Cheap, easy to design and synthesize in large quantities High tumor specificity and various of tumor antigen epitopes Regulation of immunity Low inhibitory component of tumor cells and risk of transplantation	Low immunogenicity, Weakly activated adaptive immunity, Induction of specific immune tolerance and IRAEs MHC restriction	Improve peptide/protein composition and structure Combine immune adjuvant with HSP and DC Develop individualized and even public TSA epitope peptides	Phase III/IV clinical trials: NCT00640861, NCT00640861, NCT01460472(Racotumomab)
		Cancer cell vaccines (whole cells, cell lysates and cancer stem cells)	Abundant tumor antigens Vaccine sources enriched by allogeneic tumor cells with cross-antigens Reliable clinical data analysis	High inhibitory component of tumor cells and risk of transplantation Restricted cell sources and preparation procedures Weak immunogenicity and immune tolerance	Genetic modification Develop commercial and universal allogeneic cell lines to enhance the concordance with autologous tumor antigens Improve the manufacture and use of individualized vaccines Combined with immune adjuvant	Phase III/IV clinical trials: NCT01313429, NCT00405327
	APC	DC vaccine (tumor antigen sensitization)/macrophage vaccine	High immunogenicity Abundant tumor antigens and high tumor specificity Easy to detect and control Recognition, uptake, processing, and presentation of antigens performed directly	Expensive, time-consuming, limited cell source, and requiring antigen pre-sensitization Containing many non-tumor antigens and thus inducing IRAEs;	Genetic modification Develop DC/ tumor fusion cell vaccines Combine immune adjuvant with chemotherapy, radiotherapy, and ICIs	Phase III/IV clinical trials: NCT00779402(Provenge), NCT03014804DCVax-L
	Gene transfers in vitro	Genetically modified tumor cell vaccines	Genes for MHC, costimulatory molecules, cytokines, adhesion molecules, and tumor antigens transferred to improve immunogenicity	Security risks Expensive, time-consuming, and difficult to produce	Develop the transfer and expression of individualized and even public TSAs Combine immune adjuvant with chemotherapy, radiotherapy, and ICIs	Phase I/II/III clinical trials: NCT01510288(GVAX), NCT00676507Lucanix
		Genetically modified DC vaccines (CAR-DC)	Transduction of tumor antigen genes enhancing tumor specificity, mitigating IRAEs, and addressing the limitations associated with tumor cell sources Transferring immunostimulant factor genes to augment immune responses			Phase I/II clinical trials: NCT05631886, NCT05631886
	Gene transfers in vivo	DNA/RNA vaccines (viral vaccines)	Easy to produce and safe to use Without MHC restriction Express a variety of tumor antigens easy to modified and induce long-lasting cellular and humoral immunity RNA vaccines do not easily integrate into the host genome and do not cause autoimmune diseases	Great individual variation in antigen expression, long-term low expression and immune tolerance Low immunoreactivity Unstable RNA vaccines leading to higher transport and storage conditions	Select TAAs (especially TSAs) gene with strong specificity to ensure full expression in vivo Increase cofactors to promote expression of antigen sequences	Phase I/II clinical trials: NCT02596243, NCT03164772
ACT	Unmodified cells	Lymphokine-activated killer (LAK) cells/donor lymphocyte infusion (DLI)	Enhancing cellular and humoral immunity Broad antitumor spectrum and no MHC restriction No need for antigen presensitization	Restriction of separation, purification, in vitro amplification and in vivo activity Serious IRAEs	Combine immunostimulant substances (except IL-2) Remove T cells selectively Preemptive or prophylactic DLI	LAK cells eliminated by clinical application DLI becomes the main treatment for relapsed chronic myelosuppression and EBV-associated lymphoma
		NK cells	No MHC restriction No need for antigen presensitization No serious IRAEs	Restriction of separation, purification, in vitro amplification, and in vivo activity	Optimize cell culture Combinatorial Cytokines	Phase I/II clinical trials: NCT01321008, NCT00292695
		γθT cells/cytokine-induced killer (CIK) cells/natural killer T (NKT) cells/anti-CD3 monoclonal antibody activated killer (CD3AK) cells	Enhancing cellular and humoral immunity Recognize a variety of tumor antigens No MHC restriction No serious IRAEs	Limited cell source, in vitro amplification, and cytotoxicity	CIK combined with DC, radiotherapy, chemotherapy, or BsAb Retronectin activated killer (RAK) cells	Phase I/II clinical trials: NCT04518774, NCT04292769, NCT03524261
		Tumor infiltrating lymphocytes (TILs)	High tumor specificity, low immunogenicity, and no serious IRAEs Direct and durable recognition of multiple tumor antigens	Expensive and time-consuming Limited cell sources and in vitro amplification MHC restriction Contain tumor-unrelated TILs Immunosuppressive TME	Genetic modification Select appropriate subjects Choose the high tumor-specific TIL cells (such as TDLN) Combine chemotherapy with radiotherapy and ICIs	Phase IV clinical trials: NCT05361174(Lifileucel), NCT05176470 Partial solid tumors
	Genetically engineered cells	TCR-T	High tumor specificity Identify intracellular, secretory type, lower expression, and highly mutated tumor antigens (especially TSAs) Low immunogenicity and no serious IRAEs	Expensive, time-consuming, Difficult to produce MHC restriction and recognize a limited number of antigens Off-target effects in patients with low-mutation tumors	Develop immune cells for individualized or even public TSAs Combine BsAbs to redirect ‘off-target’cells Develop commercial and universal CAR-T/NK cells using TALEN or CRISPR/cas9 novel gene editing technology (synthetic immunology) Develop specific BiCAR-NK/T Cells Combine chemotherapy, radiotherapy, and ICIs	Phase I/II clinical trials NCT00393029, NCT04520711(Partial solid tumors)
		CAR-T/CAR-γθT	No MHC restriction Recognize a variety of cell surface antigens Redirect multiple off-target T-cell subsets	Expensive, time-consuming, and difficult to produce Recognize a limited number of cell surface antigen High immunogenicity		The second-generation in clinical application(Kymriah and Yescarta) The third/fourth generation in preclinical studies: NCT05532761, NCT05640713(mainly hematological tumor)
		CAR-NK	High tumor specificity, no MHC restriction, and no severe IRAEs	Expensive, time-consuming, and difficult to produce Recognize a limited number of cell surface antigen		Preclinical studies Phase I/II clinical trials: NCT05110742, NCT05092451(mainly solid tumors)
		CAR-M	High tumor specificity, no MHC restriction, and no severe IRAE Easy to infiltrate the TME Facilitate antigen presentation and enhance T cell effects	Efficacy and safety remain to be tested clinically		Preclinical studies Phase I clinical trials (CT-0508)
Antibodies to tumor	Single -target	mAb(full length and Fab)	High tumor specificity and low side effects ADCC effect (except Fab) and CDC effect; Easy to produce and use	Tumor suppression, off-target effects, and bystander effects lead to IRAEs Hard to penetrate the cell membrane	Improve the penetration of solid tumors Overcome tumor heterogeneity Search for new specific targets Combine multiple targets with chemotherapy and radiotherapy Develop intracellular IC inhibition	Clinical application Clinical trials: NCT05941507, NCT05831878(A large proportion in cancer drug development)
	Multiple -target	BsAbs	Expand the target to overcome tumor heterogeneity and reduce side effects Redirect off-target immune cells			
	ADC		Carry cytotoxic drugs, radionuclides, or recombinant fusion proteins			

#### Induction of tumor cell death: release of internal antigens

Unlike the surface antigens of tumors, which are susceptible to the selective pressure of immunotherapy leading to drug resistance, the expression of internal antigens of tumors is more stable and closely associated with the tumor itself [167]. Although stress challenges of TME can spontaneously lead to the death of tumor cells, the artificially induced ICD of tumor cells is more conducive to the release of internal tumor antigens and the activation of immune infiltration [168]. ICD inducers have a dual effect of chemotherapy-immunotherapy. However, he effect of ICD induction in tumor cells by a single ICD inducer is usually weak and limited, which is usually achieved by triggering the secondary or “incidental” effect of anticancer drugs, and even instead promotes the formation of immunosuppressive TME [169]. On the other hand, tumors also evade ICD through various strategies, including the reduction and degradation of ATP release, the reduction of annexin A1 (ANXA1) expression, and the reduction of CRT [170]. Thus, specific interventions that are based on these defective links, such as induction of autophagy, or in combination with other immunotherapies, such as ICIs and ACT, may restore or enhance the efficacy of ICD inducers.

It is necessary to study the effect of tumor mortality and the immune effect of different ways of death [171]. According to morphological, biochemical, immunological, and genetic characteristics, regulatory cell death (RCD) can be divided into apoptotic and non-apoptotic types. Apoptosis is considered immune-tolerated, while non-apoptotic RCD can be classified as autophagy, ferroptosis, pyroptosis, and necroptosis. In some cases, non-apoptotic RCD is considered a manifestation of ICD. On one hand, autophagy can inhibit the immune escape of tumors by degrading immune checkpoints, such as the endocytic recycling of PD-L1 [172]. On the other hand, it can prevent the initiation and activation of anti-tumor T cells by mediating the degradation of MHC class I/II molecules [173]. Ferroptosis has found to enhance the anti-tumor effect of CD8 + T cells [174]. Pyroptosis and necroptosis have been shown to promote DAMP release, trigger antigen presentation, and thus activate adaptive immune responses [175]. Therefore, targeted therapies against autophagy, ferroptosis, pyroptosis, and necroptosis help to convert ICI-resistant “cold” tumors into immunologically active “hot” tumors. However, therapies targeting non-apoptotic RCD may have unintended detrimental effects on tumor-associated immune cells and lead to undesirable toxicity (Table 2) [176].

**Table 2:** ICD inducers and its mechanism of action

Classification			Molecular markers	Target	Clinical Research
Type I ICD inducers	Traditional chemotherapy agents	Anthracycline-based /DNA damage agents	ecto-CRT, ERp57, ATP, HSP70, HMGB1	Nucleus (DNA or protein involved in DNA replication) Autophagy	NCT04256616(Mitomycin CMMC)
	Targeted agents	Epidermal growth factor (EGFR) specific antibody/tyrosine kinase inhibitor (TKI)	ecto-CRT, ERp57, HSP70, HSP90	Cell surface and cytoplasm (growth signaling pathway related proteins) Pyroptosis	NCT00363649(TKI, INF-α, GM-CSF)
		Poly adenosine diphosphate-ribose polymerase (PARP) inhibitors		Nucleus (DNA repair enzyme)	NCT04675320
		Proteasome inhibitors (Bortezomib)		Cytoplasm (26 S proteasome)	NCT01472627
	Natural medicines	Cardiac glycosides	ecto-CRT, HMGB1, ATP	The cell surface (Na +, K +- ATPase)	Preclinical Studies
		Shikonin		Cytoplasm (pyruvate kinase M2, 20 s proteasome subunit)	Preclinical Studies
	Physical therapy	Radiotherapy/ultraviolet irradiation (UVC)	ecto-CRT, ERp57, ATP, HSP70, HMGB1	Nucleus (DNA or DNA replication-related proteins) Ferroptosis	NCT02921854 (Radiotherapy and cisplatin)
		High hydrostatic pressure (HHP)		Cellular proteins	Preclinical Studies
Type II ICD inducers	Physical therapy	Photodynamic therapy (PDT)/extracorporeal photochemotherapy (ECP)	ecto-CRT, ATP, ectoHSP70, HSP70, HSP90	Cytoplasm (ER, enhance intracellular oxidative stress and ER stress)	NCT05020912, NCT05157581
	Oncolytic virus (OV)	T-EVC/Coxsackie virus B3	ecto-CRT, ATP, HMGB1		Preclinical Studies
	New chemotherapeutic agents	Iridium/platinum complex	ecto-CRT, ATP, HMGB1		Preclinical Studies
Novel ICD Inducers	Physical therapy	Near infrared immunotherapy/nano pulse stimulation (NPS)	ecto-CRT, ATP, HMGB1, HSP70, HSP90	Cytoplasm (ER, enhance intracellular oxidative stress and ER stress, have tumor specificity)	Preclinical Studies
	Drug delivery	Low-temperature plasma (NTP)/nanoparticle carrier	Determined by the medication that was wrapped		Preclinical Studies
	Oncolytic peptide	LTX-315/RT53	ecto-CRT, ATP, HMGB1		NCT01223209(LTX-315)

#### Treatment strategy based on immune checkpoint

**Fig. 5:**
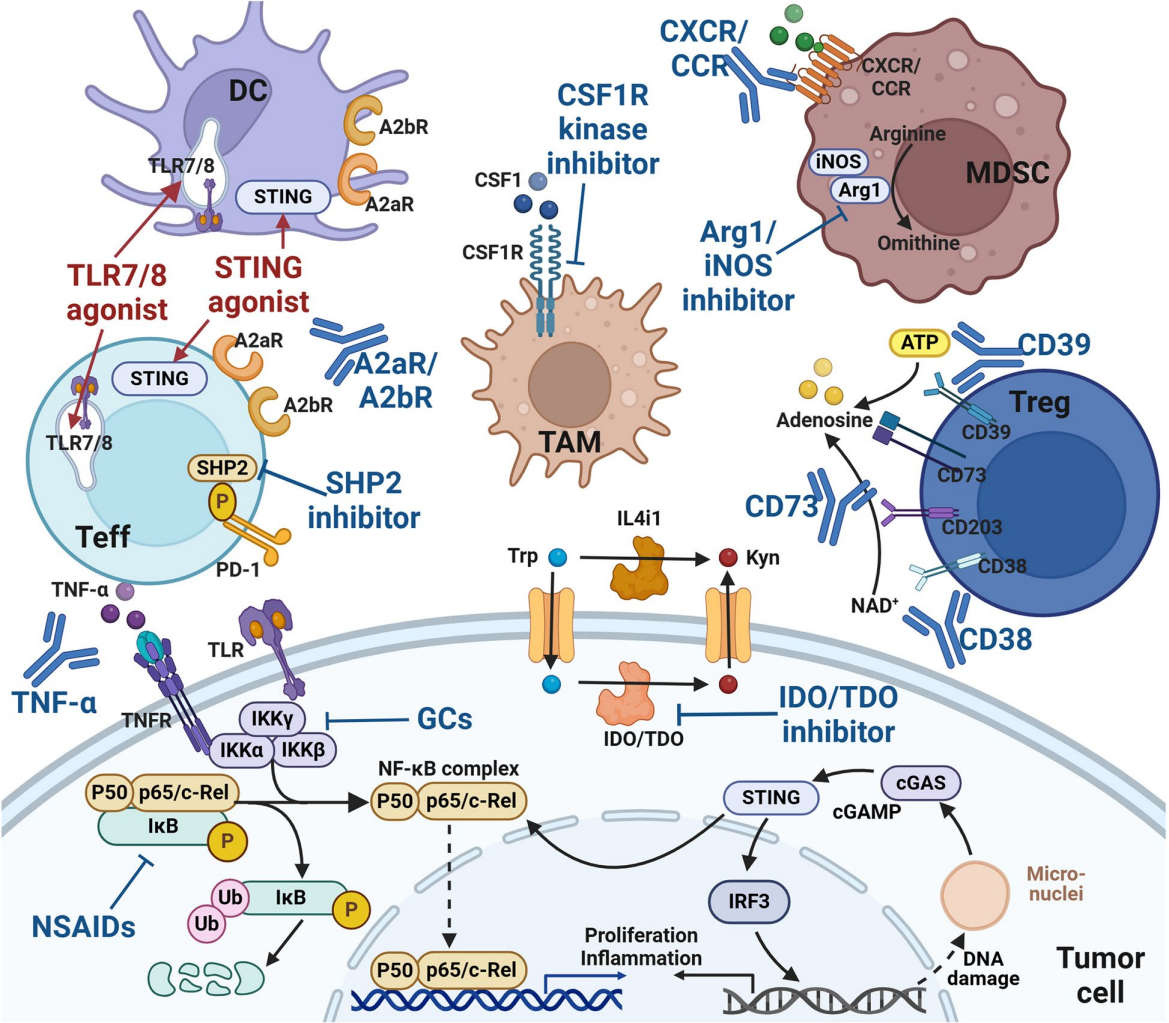
Immune checkpoint-based treatment strategies. Tumor cells actively interact with the surrounding TME and develop various adaptive strategies to form a continuously progressive and highly heterogeneous whole. Various ICs regulate tumor immune responses through a balance of inhibitory and activating signals. In addition to classical cell-surface ICs (such as PD-1/PD-L1 and CTLA-4), they also include extracellular ICs (such as TNF-α and TGF-β), intracellular ICs (such as NF-κB, STING, NR2F6 and LMTK3) and metabolism-related ICs (such as IDO and CD73). More and more ICs pathways have been found to play an important role in driving tumor immune escape. Therefore, ICS pathway may become a promising target for the development of new anticancer immunotherapy

As a crucial regulator of the immune system in healthy individuals, IC plays a pivotal role in preserving autoimmune tolerance and modulating the extent and duration of immune responses. But in the context of tumorigenesis, IC can be exploited by malignant cells, leading to aberrant expression and subsequently facilitating tumor-induced immunosuppression [177]. Therefore, the general policy of IC-based treatment strategy is to induce new or restore the original anti-tumor immune response, but it usually has problems such as low patient response rate, drug resistance and side effects. In addition to taking key IC as the main therapeutic target, other tumor immune biomarkers are also needed to predict and supervise the response of ICT. These include the gene level (such as microsatellite instability (MSI) and TMB) [178], the molecular protein level (such as IL-6, chemokines, inflammation and metabolism-related molecules [179]), the cellular level (such as immune cell frequency, TLS and TDLN), and the microbial level (such as viruses and gut microbes) [180]. In some cases, the efficacy of ICT and its side effects are complementary, that is, good efficacy is often accompanied by side effects, which in turn can be used as a sign of response to treatment [181]. Thus, how to balance ICT and its side effects deserves our further study(Fig. 5).

#### Targeting tumor-local immunity: the activation state of immune cells

A series of ICs that regulate the activation state of immune cells mainly include AKRs and IKRs of NK cells, ‘eat me’ and ‘don’t eat me’ receptors of macrophages, and positive and negative co-stimulatory molecules of T cells. In addition to classic ICs such as PD-1/L1 and CTLA-4, innate immune signals (such as TLR and STING) [182], adenosine axis signals (such as CD39 and CD73) [43], metabolic pathways of key amino acids (such as IDO) and downstream signals of TCR (such as tyrosine protein phosphatase non-receptor types (PTPN6 and PTPN22)) are all involved in the regulation of immune cell activation status [183]. It can be used as the target of novel small molecule drugs. The difference of efficacy and applicable cancer types among different ICs and the interaction between them deserve our attention.

The severity of IRAEs is contingent upon the extent of cellular involvement in ICT expression, the stage of cellular differentiation, and the underlying mechanism of action. For instance, it is noteworthy that red blood cells possess the ability to express the macrophage ‘don’t eat me’ receptor CD47, and the employment of immunotherapy targeting it may result in the development of anemia [184]. Additionally, the impact of anti-CTLA-4 primarily affects Th0 cells, potentially leading to excessive immune activation. But anti-PD-1 primarily influences Teff cells, thereby facilitating the restoration of immune-mediated anti-tumor effects [185]. Certain ICIs exhibit a more intricate mechanism of action, potentially exerting a dual effect on tumor cells. Consequently, solely blocking or enhancing their signals may not yield the desired anti-tumor outcome. For instance, as the tumor progresses, tumor cells themselves may develop tolerance to the inhibition of TGF-β, while immune cells remain suppressed due to the abundant secretion of TGF-β. This immune escape of tumors implies that restoring the sensitivity of tumor cells to TGF-β could prove more beneficial in addressing the challenges encountered in TGF-β antibody therapy [186]. In addition, relevant studies on the endocytosis, recycling, and degradation mechanism of PD-L1 help to make up for the deficiency of PD-L1 antibody therapy (Table 3) [187].

**Table 3:** Immune checkpoint-based therapeutic strategies

Classification		Receptors (immune cells)	Distribution	Ligand (tumor cells)	Characteristics	Clinical Application
NK cells	IKRs	KIR2DL/3DL(CD158)	NK, T, NKT cells	Classical MHC-I molecules(HLA-A/B/C)	IgSF includes KIR, CD96, LIR and Siglec Have a significant prevalence of genetic polymorphisms and ethnic disparities, associated with disease susceptibility, particularly autoimmune disorders Identify small MHC-I changes	Anti-KIR: Lirilumab/IPH2102, Lacutamab/IPH4102 Leukemia, MM, SCCHN, BC, ovarian cancer NCT2252263
		NKG2A/B(CD159)	NK (CD56hi), NKT, T cell (αβT)	Non-classical MHC-I molecules(HLA-E)	Belong to the C-type lectin family and form a heterodimer with CD94 Non-genetic polymorphism, associated with GVHD and autoimmune diseases	Anti-NKG2A: Monalizumab/IPH2201 CLL, BC, ovarian cancer, SCCHN, CRC, NSCLC NCT02459301
		CD96	NK, T cells	CD155	CD96, TIGIT (suppress) and CD226 (stimulus) combined with CD155 competitively Have a costimulatory effect	CD96 inhibitor: Preclinical studyies HCC, colorectal cancer
		LIRs(LILR, ILTs)	NK, T, B cells, TAM, DC, MDSCs	HAL-G	Classified into activating (LIRA1-6) and inhibitory (LIRB1-5) receptors, which inhibit cytotoxicity, cytokine (IFN-γ) and chemokine secretion	Anti - LIR: BND − 22/SAR444881 Leukemia, HCC, RCC, NSCLC NCT04717375
		Siglec-7/9 (CD33-related)	NK, B, T cells, DC, TAM	Sialoglycan	Classified into inhibitory, activating, and non-signaling receptors The expression and modification of Siglec and its ligands are diverse Associated with GVHD and autoimmune diseases	Anti-Siglec: mAb or ADC Preclinical studyies Anti-Sialoglycan: E-602 double sialidase NSCLC, CRC, BC NCT05259696
		CD200R	T, B, NK cells, TAM	CD200 (T, B cells (activating), DC, normal tissue cells)	Inhibit the expression of TNF-α, IFN-γ and iNOS, and promote peripheral immune tolerance Have a contradictory effect The degree of infiltration of CD200 + T cells can be used as a marker of the efficacy of ICI	Anti-CD200: samalizumab Leukemia NCT05512104 Activate CD200: CD200AR -l Glioblastoma NCT04642937
	AKRs	KIR2DS/3DS	NK, T, NKT cells	Classical MHC-I molecules	Inhibitory KIR (KIR2DL/3DL), activating KIR (KIR2DS/3DS), and dual signaling KIR( KIR2DL4/CD158d)	Activate KIR: NK cell transplantation Leukemia, lymphoma, hemangioma NCT01875601
		NKG2C NKG2D(CD314)/ NKG2E	NK (CD56hi), NKT	HLA-E HLA-A/B, ULBP	Inhibitory (NKG2A/B) and activating (NKG2C/D/E) receptors. All except NKG2D formed heterodimers with CD94 NKG2D/E homology is high, and NKG2C competes with NKG2A for HLA-E	Genetically modified NKG: CAR-NK cells Leukemia and ovarian cancer NCT05776355
		NCRs: NKp30/44/46 NKp80	NK cells	Viral HA, B7-H6, BAG6 AICL	NK cell-specific markers (NKp44 is a marker of activating NK) Tumor-associated ligands are mostly unknown	Connecting and activating NKp46: IPH6101/SAR443579 Leukemia NCT05086315
		DNAM-1 (CD226)	NK, T cells	CD112 (Nectin-2), CD155 (PVR)	Belong to Nectin/Necl family, both CD226 and TIGIT bind to CD112 and CD155 CD226 promotes NK cells from quiescence to activation, while TIGIT negatively regulates NK cells after activation.	Activate CD226: preclinical studyies
TAM	Don’t eat me	SIRPα	TAM, nerve cells, DC	CD47	CD47 is widely expressed in normal cells (especially the RBC and T cell), also combined with integrin and TSP – 1; associated with proinflammatory response and antigen presentation	Anti-CD47: Magrolimab Leukemia, brain tumor, CRC NCT03248479
		CSF1R	Monocyte-macrophage, osteoclasts	CSF-1 (M-CSF)	Tyrosine-specific protein kinase activity Promote the differentiation of TAM and MDSCs, but inhibite the maturation of DCs maintain the polarization of M2	Anti-CSF-1: CSF1R-TKI (PLX3397) or mAb (RG7155) TGCT, BC, prostate cancer, pancreatic cancer, renal cancer NCT01494688
	Eat me	TLR1 TLR2/4/5 TLR3	Monocyte-macrophages, T, B, NK cells, DC, tumor cells	DAMPs, PAMPs	Ligands are widely recognized to activate innate and adaptive immunity Mediate the production of cytokines (INF-α) to regulate immunity	Activate TLR: TLR agonists (ligands) or immune adjuvants Melanoma, BC, bladder cancer, cervical cancer NCT02668770
T cells	Co-inhibitory molecules	PD-1 (CD279)	T, NK, B cells, DC, TAM	PD-L1 (B7-H1), PD-L2 (B7-DC)	Promote the apoptosis of CD8 + T cells and reduce the apoptosis of Treg cells in the late stages of the immune responselate in the immune response PD-L1 is widely distributed, heterogeneous and dynamically changing	Anti-PD-1/PD-L1: Pembrolizumab, Atezolizumab Melanoma, NSCLC, gastrointestinal cancer, BC, leukemia NCT04333004
		CTLA-4 (CD152)	T cells	CD80/CD86	Competitively inhibit CD28 mainly in the early TDLN Inhibit the immune response of T cells Lead to severe IRAEs and related to autoimmune diseases Mediate the transcytosis of APC ligands	Anti-CTLA-4: ONC-392 (the second generation) Gastric cancer, HCC, NSCLC, melanoma NCT05671510
		LAG-3 (CD223)	T, NKT, NK, B cells, pDC, neurons	MHC II, FGL1, galectin-3, LSECtin	Similar to the structure of CD4 Inhibit both CD8 + T and Treg cell activity Co-expressed with PD-1 and functionally complementary	Anti-LAG-3: Relatlimab NSCLC, melanoma, esophageal cancer, gastric cancer, and leukemia NCT05498480
		Tim-3 (CD366/ HAVCR2)	T, NK cells, TAM, DC	Galectin-9, CEACAM-1, HMGB1, PtdSer	Play an activating role when not bound to the ligand but an inhibitory role when bound to the ligand Associated with autoimmune diseases Co-expression with PD-1	Anti-Tim-3: IBI104 (endocytosis) NSCLC, CRC, HCC, Gastric cancer, leukemia NCT03568539
		TIGIT	T, NK cells	PVR family: CD155 CD112, CD113	CD155 and CD112 are ligands for CD226 (costimulatory molecule) and CD96 (coinhibitory molecule); co-expressed with PD-1, TIM-3 and LAG-3	Anti-TIGIT: Tiragolumab or combined with PD-1/ PD-L1; lung cancer, cervical cancer, melanoma, gastroesophageal cancer NCT05715281
		VISTA (PD-1 H or DD1α)	T cells, plasma cells, neutrophils, DC, TAM, MDSCs	VSIG3 (IgSF11), PSGL 1 (pH dependent)	Similar to the structure of PD-L1, but its inhibitory function is independent Constitutively expressed in naive T cells to maintain quiescence Associated with autoimmune diseases	Anti-VISTA: KVA12123 NSCLC, CRC, TNBC, RCC NCT05708950
		BTLA	T, B, and NK cells, DC, TAM	HVEM (TNFRSF14, CD270)	Like the structure and function of PD-1 and CTLA-4 Bind of HVEM to BTLA/CD160 triggers a coinhibitory signal, while bind of HVEM to LIGHT/LTα transmits a costimulatory signal	Anti-BTLA: AVTX-002, JS004 NSCLC, melanoma, lymphoma, HNSCC NCT02857166
		TLT-2 (mouse); unknown	T, NK, B cells, DC	B7-H3(CD276) B7-H4(B7x)	B7 family includes B7-1 (CD80) and B7-2 (CD86), B7-H1 (PD-L1) and B7-DC (PD-L2), B7-H3 and B7-H4 Dual functions of activation and inhibition	Anti-B7-H3/H4: mAb, ADC, CAR-T Prostate cancer, BC, bladder cancer, NSCLC NCT04432649
	Costimulatory molecule	CD28	T cells	CD80 (B7-1)/CD86 (B7-2)	IgSF includes CD28, ICOS, CD266, CTLA4 and PD1 Enhanced by CD40 signaling Form positive feedback with TCR and Ca2+ Associated with autoimmune diseases	Activate CD28: CD28 agonist (CD80 fusion protein) Leukemia, lymphoma, ovarian cancer, BC NCT00603460
		OX40 (CD134)	T, NK, NKT cells (constitutively expressed on Treg, inductively expressed on activating Teff)	OX40L (CD252, expressed in APC, NK, hypertrophy and activated of T cells)	TNFRSF includes OX40, CD40, CD27, 4-1BB, CD270 and GITR Enhance TCR signaling and attenuat CTLA-4 and Foxp3 signaling Activate NF-κB, PI3k, and NFAT pathways	Activate OX40: OX40 agonist (agonist Ab) Leukemia, CRC, BC, RCC, melanoma NCT03092856
		ICOS (CD278)	T cells (activating)	ICOSL (B7-H2, B cell, TAM, DC)	Not only promote the occurrence of tumors, but also have synergistic effect with CLTA-4 Highly homologous to B7-1/2 and affect Tm and Teff	Activate ICOS: GSK3359609 HNSCC NCT03693612 Anti-ICOS: MEDI-570 Lymphoma NCT02520791
		4-1BB (CD137)	T, B, NK cells, DC, tumor cells	4-1BBL (B, T cell (activating), TAM, DC)	NF-κB, AKT, p38 MAPK and ERK pathways are used to induce the expression of anti-apoptotic genes and reduce the expression of pro-apoptotic genes	Activate 4-1BB: CAR-T or 4-1BB agonist (agonist Ab) Lymphoma, leukemia, NSCLC, Gastric cancer, BC NCT05040932
		CD27	T, B, NK cells	CD70 (DC, B, T, NK cells, upon activated, constitutively expressed)	Induce proliferation and differentiation of Tm and Teff, and enhance the activation of B and NK cells Have synergistic effect with CD28	Activate CD27: CD27 agonist (agonist Ab) Leukemia, NSCLC, melanoma, RCC NCT01460134
		CD40L (CD154)	T, B, NK cells (activating)	CD40 (DC, B cells, TAM, platelet)	Promote the formation of immune synapses between APC and T cells, upregulate the expression of MHC and CD86/CD80 Associated with autoimmune diseases	Activate CD40: CD40 agonist (excited Ab) Melanoma, colon cancer, Bladder cancer, leukemia NCT04635995
		GITR (CD357)	T, NK cells, keratinocytes, osteoclasts	GITRL (DC, B, TAM, endothelial cells)	Different from the “atypical” interaction mode of classical TNF/TNFRSF	Activate GITR: GITR agonist (agonist Ab/ligand) Melanoma, HNSCC, NSCLC, HCC NCT02598960
Intracellular IC		NR2F6 (EAR2)	Almost all cells (particularly lymphocytes)	Unknown	Intrinsic orphan nuclear receptors Negatively regulate the activation signal of TCR/CD28 and inhibit the production of cytokines (IL-2, TNF-α and IFN-γ) Associated with pro/anti-inflammatory balance, cell differentiation, and cell fate	Anti-NR2F6: circRHOT1, miR-142-3p Leukemia, lymphoma, NSCLC, BC, CRC Preclinical studies
		STING	Tumor, NK, B cells, DC, TAM	Abnormal DNA	cGAS-STING signaling pathway Have a dual role in inhibiting/promoting cancer	Activate STING: STING agonist Lymphoma, leukemia, HCC, BC, stomach cancer, esophageal cancer NCT04144140
		LMTK3	Tumor cells	HSP90	Oncogenes, active protein kinases of oncogenic regulators, require HSP90 for folding and stabilization Regulate ER activity Associated with endocrine resistance, chemotherapy resistance, and CNS disease	Anti-LMTK3: small molecule inhibitor C28 BC, stomach cancer, NSCLC, CRC, bladder cancer Preclinical studies
Extracellular IC		TGF-β	T, B, and NK cells, TAM, DC	TβR	Dual effects of inhibiting/promoting cancer	Anti-TGF-β: blocking antibodies, TGF-β trap or small molecule inhibitors Melanoma, bladder cancer, esophageal cancer, sarcoma, pancreatic cancer NCT05537051
		TNF-α	Monocyte-macrophages, neutrophils, T, NK, tumor cells	TNFR - I (activation key)/TNFR - II (high affinity, immune cells)	Ligand of TNF superfamily Associated with inflammation, immune system development, apoptosis, and lipid metabolism Have dual effects of inhibiting/promoting cancer	Activate TNF-α: rhTNF-α combined with liposomal doxorubicin, Advanced solid tumors and lymphoma NCT01490047 Anti-TNF-α: mesna/Icaritin HCC NCT01205503
Metabolism related IC		IDO	Tumor cells	None	An endogenous rate-limiting enzyme in the Trp degradation to Kyn pathway Associated with nerve repair	Anti-IDO: small molecule inhibitors, IDO vaccine Leukemia, melanoma, prostate cancer, pancreatic cancer, BC NCT01792050
		CD73/CD38/CD39/A2aR/A2bR	T, NK cells, DC, TAM, MDSCs	ATP/adenosine	Innate negative feedback mechanisms of the adenosineergic axis	Anti-CD73/CD38/CD39/A2Ar/A2bR: mAb or small molecule inhibitors or CAR - T MM, HCC, NSCLC NCT04797468, NCT05239689

#### Targeted tumor system immunity: development, differentiation and recruitment of immune cells

Tumor as a systemic disease can remodel function and composition of peripheral immune cells. It is mainly manifested by changes in the frequency of immune cells and their related factors in blood circulation and immune organs, resulting in different responses to immunotherapy in patients with different immune backgrounds [72]. Tumors have the capacity to remodel the microenvironment of immune organs, primarily bone marrow and TDLN, thereby impeding the maturation of immune cells and promoting the development of immunosuppressive phenotypes [188]. Additionally, tumors can modulate the expression of chemokines in the bloodstream, leading to the suppression of anti-tumor immune cells while facilitating the migration of pro-tumor immune cells from immune organs to the TME [189]. In addition to immunotherapy, traditional tumor treatments such as surgery, radiotherapy and chemotherapy can also change the immune background environment of the tumor and affect the therapeutic effect [72]. Therefore, the general principle of therapeutic strategies targeting tumor systemic immunity is to promote the development, differentiation and recruitment of anti-tumor immune cells or inhibit them of pro-tumor immune cells. However, the specificity and accuracy of artificial regulation of tumor systemic immunity are low (except for targeting colony-stimulating factor 1 (CSF-1) [190]). At present, it is mainly used to monitor the progress of tumor immunity and predict the effect of immunotherapy by detecting tumor systemic immunity [191].

## Conclusions

In the current review, we comprehensively summarized the war between the immune system and the tumors. As a large battle, ICs provide the necessary trace for the immune system and drugs to accurately attack the tumor. Interestingly, emerging factors significantly affect tumor immunity and thus mediate immunotherapy responses. As a form of physiological intervention, exercise reduces cancer risk and cancer-related morbidity, also re-shape TME via several machineries, should deserve more attentions in future research. Given the complex and diverse regulatory mechanisms of anti-tumor immunity, it is certain that an improved understanding of the unbalance between the immune system and the tumors will provide useful insights into tumorigenicity and may lead to novel clinical applications.

## Data Availability

No datasets were generated or analysed during the current study.

## Abbreviations

ACT: Adoptive cell therapy
ADC: Antibody drug conjugate
ADCC: Antibody-dependent cell-mediated cytotoxic effect
ADCP: Antibody-dependent cell-mediated phagocytos
AICD: Activation induced cell death
AKR: Activated killer cell receptor
ANXA1: Annexin A1
APC: Antigen presenting cell
Arg-1: Arginase 1
ATP: Adenosine triphosphate
Bregs: Regulatory B cells
BsAbs: Bispecific antibodies
CAFs: Cancer-associated fibroblasts
CAR: Cchimeric antigen receptor
CDC: Ccomplement-dependent cytotoxicity
cDC1: Type 1 conventional dendritic cells
cDC2: Type 2 conventional dendritic cells
CRC: Colorectal cancer
CRT/CALR: Calreticulin
CSDE1: Cold shock domain-containing protein E1
CSF-1: Colony-stimulating factor 1
CTL: Cytotoxic T lymphocyte
CTLA-4: Cytotoxic T lymphocyte antigen 4
DAMPs: Damage-associated molecular patterns
DAR: Drug antibody coupling ratio
DCs: Dendritic cells
ECM: Extracellular matrix
EDMCs: Erythroid derived myeloid cells
EPCs: Erythroid progenitor cells
ER: Endoplasmic reticulum
FAO: Fatty acid oxidation
Gln: Glutamine
Her-2: Human epidermal growth factor receptor 2
HERVs: Human endogenous retroviruses
HLA: Human leukocyte antigen
HMGB1: High mobility group protein 1
HSP: Heat shock protein
ICIs: Immune checkpoint inhibitors
ICs: Immune checkpoints
ICT: Immune checkpoint therapy
IDO: Indoleamine 2, 3-dioxygenase
IFN-γ: Interferon-γ
IKRs: Inhibitory killer cell receptors
IL-10: Interleukin-10
iNOS: Inducible nitric oxide synthase
IRAEs: Immune-related adverse events
IRF1: interferon regulatory factor 1
KLF2: Kruppel-like factor 2
Kyn: Kynurenine
lncRNA: Long non-coding RNA
mAbs: Monoclonal antibodies
MAPK: Mitogen-activated protein kinase
MDSCs: Marrow-derived suppressor cells
MHC: Major histocompatibility complex
M-MDSCs: Monocyte MDSCs
mregDC: Mature regulatory dendritic cell
MSI: Microsatellite instability
MSLN: Mesothelin
NK cells: Natural killer cells
NR2F6: Nuclear receptor subfamily Group 2 F-member 6
PD-1: Programmed death receptor-1
PGE2: Prostaglandin E2
pMHC: Peptide-MHC
PMN-MDSCs: Polymorphonuclear cells MDSCs
PRRs: Pattern recognition receptors
RCD: Regulatory cell death
SASP: Senescence-associated secretory phenotype
sPD-L1: Soluble PD-L1
TAAs: Tumor-associated antigens
TAMs: Tumor-associated macrophages
TCRs: T cell receptors
TDLN: Tumor draining lymph node
Teff: Effector T cells
Tex: T cell exhaustion
TGF-β: Transforming growth factor β
Th0: Initial T cells
TLRs: Toll-like receptors
TLS: Tertiary lymphoid structure
TMB: Tumor mutation burden
TME: Tumor microenvironment
TNF: Tumor necrosis factor
TRAIL: TNF-related apoptosis-inducing ligand
Tregs: Regulatory T cells
TRM: Tissue resident memory T cells
Trp: Tryptophan
TSAs: Tumor-specific antigens
VEGF: Vascular endothelial growth factor
VISTA: V-domain Ig suppressor of T cell activation
VM: Vasculogenic mimicry
β2m: β2 microglobulin
